# Transcriptome characterization of gonadal sex differentiation in Pacific bluefin tuna, *Thunnus orientalis* (Temminck et Schlegel)

**DOI:** 10.1038/s41598-023-40914-y

**Published:** 2023-08-24

**Authors:** Takao Hayashida, Satoshi Soma, Yoji Nakamura, Kentaro Higuchi, Yukinori Kazeto, Koichiro Gen

**Affiliations:** 1 Nagasaki Field Station, Fisheries Technology Institute, Japan Fisheries Research and Education Agency, 1551-8 Taira-machi, Nagasaki, Nagasaki, 851-2213 Japan; 2 Graduate School of Marine Science and Technology, Tokyo University of Marine Science and Technology, 4-5-7 Konan, Minato-ku, Tokyo, 108-8477 Japan; 3 Yokohama Field Station, Fisheries Resources Institute, Japan Fisheries Research and Education Agency, 2-12-4 Fuku-ura, Yokohama, Kanagawa 236-8648 Japan; 4 Minamiizu Field Station, Fisheries Technology Institute, Japan Fisheries Research and Education Agency, 183-2 Minamiizu, Kamo, Shizuoka, 415-0156 Japan

**Keywords:** Developmental biology, Ecology

## Abstract

Tunas (genus *Thunnus*) are one of the most ecologically and commercially important fish worldwide. To establish a biological basis for reproduction in this globally essential species, we have recently studied crucial reproductive aspects of the Pacific bluefin tuna (*T. orientalis*; PBT), as a model of tuna species, based on our closed-cycle aquaculture technology. In this study, we clarified the global expression profile of the genes regulating gonadal sex differentiation in PBT, as this developmental process is vital to sexual reproduction. Based on the results of our comparative (RNA-sequencing) and temporal (qRT-PCR) transcriptome analyses using the updated genome dataset, we propose the molecular mechanisms of gonadal sex differentiation in PBT. In female gonads, *foxl2* and *cyp19a1a* (coding aromatase) are expressed at the onset of sex differentiation. Active aromatase-mediated estrogen biosynthesis, which includes positive regulation of *cyp19a1a* expression by Foxl2, induces ovarian differentiation. By contrast, *dmrt1* and *gsdf* are upregulated in differentiating male gonads lacking active estrogen synthesis. Dmrt1 and Gsdf would mainly promote testicular differentiation. Furthermore, androgen biosynthesis is upregulated in differentiating male gonad. Endogenous androgens may also be vital to testicular differentiation. This study provides the first comprehensive data clarifying the molecular basis for gonadal sex differentiation in tunas.

## Introduction

Tunas (*Thunnus* spp.) are widely distributed in the global ocean and are top predators in marine ecosystems. Tuna, specifically bluefin tuna, has a high market demand and value and is of substantial commercial interest to fisheries and aquaculture operations worldwide. Despite the ecological and commercial importance of tuna, the mechanisms by which its reproductive system develops remain poorly understood, mainly owing to the difficulty in obtaining artificial seedlings that can be used for experiments and their rearing^1^. A detailed understanding of the biological basis for tuna reproduction is necessary to achieve several goals in fisheries and aquaculture operations, such as effective assessments of population fluctuations in wild-stock management^2^ and efficient broodstock management in captivity to obtain high-quality fertilized eggs^3,4^. Hence, we recently studied the vital reproductive aspects of Pacific bluefin tuna (*T. orientalis*; hereafter, PBT), as a model of tuna species, based on our closed-cycle aquaculture technology^1,5–8^.

Gonadal sex differentiation is crucial in vertebrate sexual reproduction. In gonochoristic fishes (many species of fish, including PBT^6^, are gonochoristic^9^), gonadal primordia are formed at the early embryonic stage and subsequently differentiate into ovaries or testes under the control of various genes and factors^10^. Estrogens are critical factors in fish ovarian differentiation^10–12^. *Cyp19a1a* (cytochrome P450 aromatase, P450-arom; hereafter, aromatase) is expressed mainly in the female gonads at the onset of sex differentiation, and estrogens synthesized by aromatase directly induce ovarian differentiation^10–12^. The transcription factor forkhead box protein L2 (Foxl2) is also a major player in active estrogen biosynthesis as it facilitates *aromatase* expression^11,12^. By contrast, the physiological roles of androgens in testicular differentiation vary among fish species. For instance, androgen production is absent in the male gonads of Nile tilapia (*Oreochromis niloticus*) during testicular differentiation^13,14^. Moreover, androgens are actively synthesized in the differentiating male gonads of rainbow trout (*Oncorhynchus mykiss*)^15,16^. Doublesex and mab-3 related transcription factor 1 (Dmrt1) is highly conserved and orchestrates testicular differentiation in various different vertebrates^10^. Dmrt1 facilitates the expression of genes regulating testicular differentiation, such as *amh* (*anti-Müllerian hormone*) in zebrafish (*Danio rerio*)^17^ and *sox9b* (*SRY-box transcription factor 9b*) in Nile tilapia^18^. Dmrt1 may also directly block estrogen production as reported in Nile tilapia^19^. The gonadal soma-derived factor (Gsdf) also induces testicular differentiation in fish^10^. *Gsdf* upregulation in differentiating male gonads has been confirmed for various fish species^10^. The vital role of Gsdf in testicular differentiation has been demonstrated through loss- and/or gain-of-function studies in several fish species including Nile tilapia^20^ and medaka (*Oryzias latipes*)^21^.

We recently demonstrated the timing and morphological process of gonadal sex differentiation in PBT^6^. Furthermore, no difference in germ cell proliferation and differentiation between sexes during gonadal sex differentiation was observed^6^. We also showed the vital role of aromatase-mediated estrogen biosynthesis in ovarian differentiation in PBT. Aromatase inhibitor (AI) administration resulted in sex reversal of genotypic female to phenotypic male^1^. We confirmed testicular differentiation of the AI-treated genotypic female PBT gonads at the molecular level through histological observation, gene expression analysis, and serum sex steroid measurement^1^. However, the detailed molecular mechanisms of gonadal sex differentiation in general, and testicular differentiation in particular, remain unclear. A detailed understanding of the gonadal sex differentiation mechanisms will provide a fundamental basis for developing reproductive biotechnology in aquaculture, specifically sex-manipulation technologies^22^.

Application of next-generation sequencing (NGS) technology has recently become increasingly common in the field of aquaculture and fisheries. High-throughput RNA sequencing (RNA-Seq) technology is effective in studying the molecular mechanisms underlying the various biological characteristics of non-model and model species and has high reproducibility. The objective of the present study was to elucidate the molecular mechanisms of gonadal sex differentiation in PBT using NGS technologies. Our research group published the first-draft PBT genome in 2013^23^ and improved its assembly in 2019^25^ and again in 2021^25^. In this study, we initially polished the 2021 version of our PBT draft genome assembly^25^ and predicted the protein coding sequences using the refined genome. Using the predicted gene dataset as a reference, we then examined the global gene expression profiles of gonadal sex differentiation in PBT via comparative transcriptome analysis based on RNA-Seq technology. Furthermore, we examined the temporal expression profiles of the candidate genes controlling gonadal sex differentiation using quantitative real-time reverse transcription PCR (qRT-PCR).

## Results

### Reference gene dataset construction using a polished draft genome

The 2021 version of the PBT draft genome^25^ was rearranged from 948 to 580 scaffolds by polishing the sequence and eliminating redundancy. The completeness score obtained via Benchmarking Universal Single-Copy Orthologs (BUSCO) assessment, i.e., the reference ortholog capture ratio of the older version was 98.2%, of which 95.7% were singletons and 2.5% were duplicated^25^. This ratio was 98.4% for the current version, of which 97.7% were singletons and 0.7% were duplicated. Few of the doubly predicted orthologs in the previous version (~ 2% of 3640 reference genes) ^25^ were predicted as singletons by removing redundant scaffolds. We predicted 30,156 protein-coding sequences from the current PBT genome (Supplementary Data 1). Of these, ~ 90% resembled those corresponding to the zebrafish and medaka protein sequences (see Supplementary Table S1 online).

### RNA-sequencing and comparative transcriptome analyses

Table 1 summarizes the RNA sequencing results. Sequencing of the cDNA libraries yielded 75–102 million paired-end reads (75 bp). Over 99% of the sequenced reads were clean following quality control. Clean reads (89.9–90.7%) were read-mapped to the reference sequences.

**Table 1:** Results of RNA sequencing.

Sample	RNA integrity no. (RIN)	No. reads	No. clean reads	No. clean bases (Gb)	GC content (%)	Total No. mapped reads (%)
Female-1	9.2	79,685,638	79,682,070	5.21	47.6	90.7
Female-2	8.9	75,238,374	75,234,984	4.92	47.3	90.7
Female-3	8.3	102,356,556	102,352,454	6.70	47.0	90.3
Male-1	7.3	81,855,526	81,852,002	5.36	46.9	89.9
Male-2	8.2	77,421,316	77,417,522	5.07	46.7	90.6
Male-3	7.7	78,289,102	78,285,284	5.12	46.9	90.1

**Figure 1:**
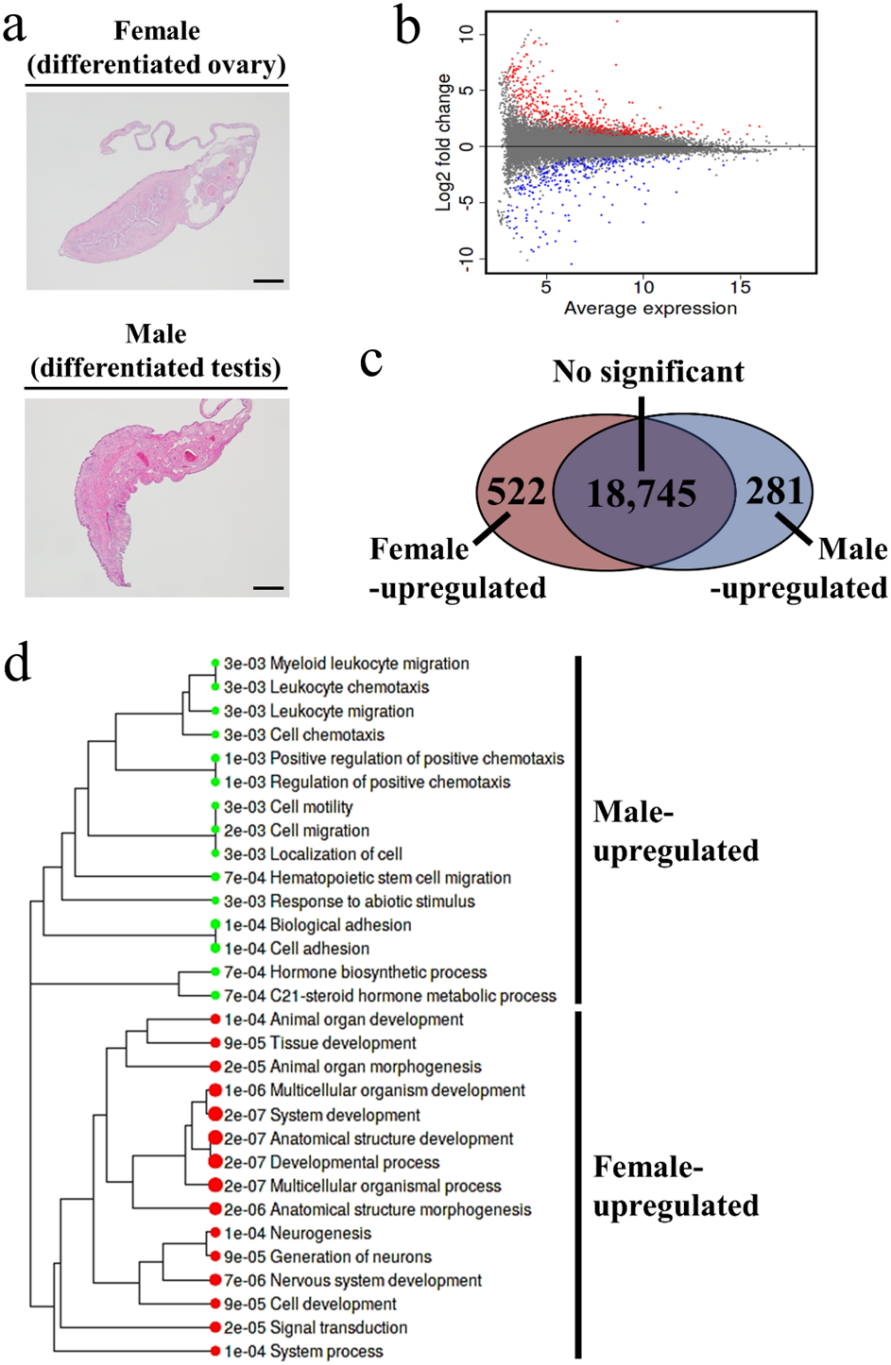
Summary of comparative transcriptome analysis. (**a**) Histological micrographs of sex-differentiated stage Pacific bluefin tuna gonads subjected to RNA sequencing. Bars = 200 µm. (**b**) MA plot showing differentially expressed genes. Significantly upregulated (*P* < 0.05) genes in females and males are indicated by red and blue dots, respectively. Genes not significantly different between sexes (*P* > 0.05) are indicated by gray dots. (**c**) Venn diagram showing numbers of commonly (*P* > 0.05) and differentially (*P* < 0.05) expressed genes between sexes. (**d**) Hierarchical clustering tree showing the top 15 enriched Gene Ontology (GO) biological process terms in upregulated genes for each sex. The circle size and number on left side of each GO terms indicate false discovery rate adjusted *P*-values.

The PBT gonads at the sex-differentiated stage (Fig. 1a) were subjected to comparative transcriptome analysis (Fig. 1b,c). A total of 522 and 281 genes were significantly upregulated in the females and males, respectively (*P* < 0.05, see Supplementary Table S2 online). Both sexes shared the expression of 18,745 genes (*P* > 0.05, see Supplementary Table S3 online). Figure 1d and Supplementary Tables S4–S5 show the top 15 Gene Ontology (GO) biological process terms enriched in each sex from the genes differentially expressed between sexes. In the females, several cell/tissue development-related terms, including ‘animal organ development’ (GO: 0,048,513), ‘tissue development’ (GO: 0,009,888), ‘animal organ morphogenesis’ (GO: 0,009,887), ‘multicellular organism development’ (GO: 0,007,275), ‘system development’ (GO: 0,048,731), ‘anatomical structure development’ (GO: 0,048,856), ‘developmental process’ (GO: 0,032,502), ‘multicellular organismal process’ (GO: 0,032,501), ‘anatomical structure morphogenesis’ (GO: 0,009,653), ‘cell development’ (GO: 0,048,468), and ‘system development’ (GO: 0,003,008) were detected. In the males, structural development-related terms such as ‘cell motility’ (GO: 0,048,870), ‘cell migration’ (GO: 0,016,477), ‘localization of cell’ (GO: 0,051,674), ‘biological adhesion’ (GO: 0,022,610), and ‘cell adhesion’ (GO: 0,007,155) were detected. The steroidogenesis-related terms ‘hormone biosynthetic process’ (GO: 0,042,446) and ‘C21-steroid hormone metabolic process’ (GO: 0,008,207) were also detected in the males.

**Figure 2:**
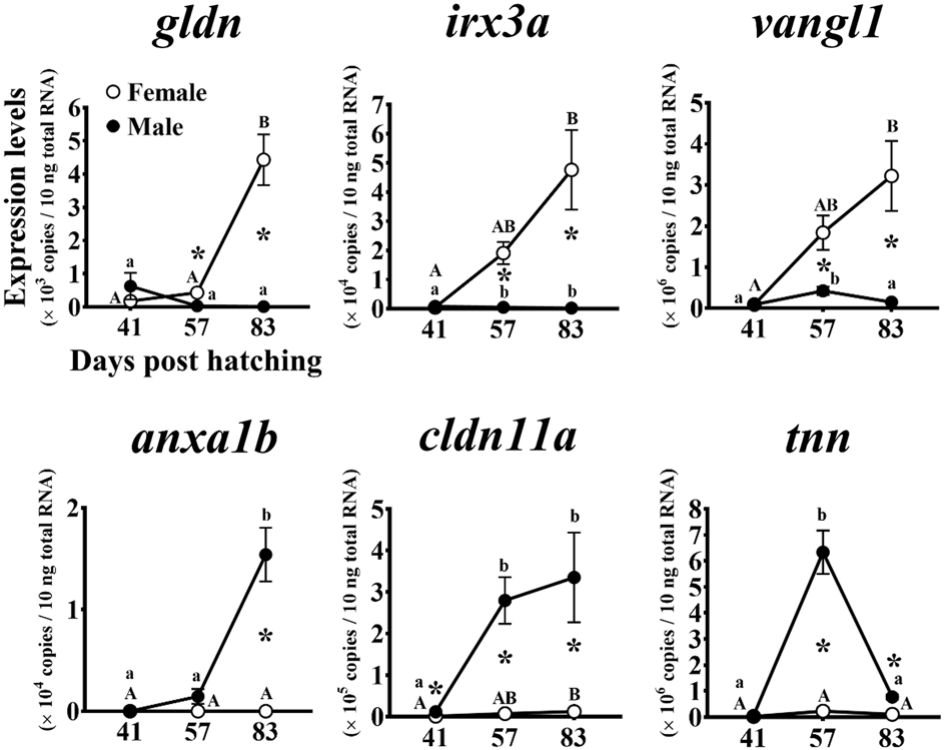
Temporal expression patterns of genes encoding unreported factors in fish sex differentiation during gonadal sex differentiation in Pacific bluefin tuna. Quantitative real-time reverse transcription (qRT-PCR) targeted three genes for each sex which were enriched in multiple Gene Ontology (GO) pathways (*gldn*, *irx3a*, and *vangl1* in females, and *anxa1b*, *cldn11a*, and *tnn* in males; see Supplementary Table S4–S5 online). Total RNA extracted from the gonads of each sex at morphologically sex-undifferentiated (41 days post-hatching (dph)), differentiating (57 dph), and differentiated (83 dph) stages^6^ were subjected to qRT-PCR. Open and closed circles indicate genotypic females and males, respectively. Bars indicate the mean ± standard error of the mean (SEM) (*n* = 3 fish). Different letters indicate significant differences (*P* < 0.05, one-way ANOVA followed by Tukey’s multiple comparisons test). Asterisks indicate significant differences between sexes at each age (*P* < 0.05, Welch’s *t*-test).

The expression profile of the genes enriched in several GO terms was exemplary validated using qRT-PCR (Fig. 2). The qRT-PCR targeted three upregulated genes for each sex: *gldn* (*gliomedin*; contig No. g2699), *irx3a* (*iroquois homeobox 3a*; contig No. g3224), and *vangl1* (*VANGL planar cell polarity protein 1*; contig No. g5686) in female, and *anxa1b* (*annexin A1b*; contig No. g5180), *cldn11a* (*claudin 11a*; contig No. g10146), and *tnn* (*tenascin N*; contig No. g11982) in males (see Supplementary Table S4–S5 online). Furthermore, their temporal expression during gonadal sex differentiation was identified. In the females, the *gldn*, *irx3a*, and *vangl1* expressions significantly increased from the morphologically sex-undifferentiated (41 days post hatching) to the differentiated (83 dph) stages (*P* < 0.05). However, in the males, the *gldn*, *irx3a*, and *vangl1* expressions remained low until the differentiated stage. The *gldn*, *irx3a*, and *vangl1* expressions were significantly higher in the females than in the males at the differentiating (57 dph) and differentiated (83 dph) stages (*P* < 0.05). In the males, the *anxa1b* and *cldn11a* expressions significantly increased from the undifferentiated (41 dph) to the differentiated (83 dph) stages (*P* < 0.05). The *cldn11a* and *tnn* expressions significantly increased from the undifferentiated (41 dph) to the differentiating (57 dph) stages (*P* < 0.05). The *anxa1b*, *cldn11a*, and *tnn* expressions remained low until the differentiated stage in the female. The *anxa1b*, *cldn11a*, and *tnn* expressions were significantly higher in the males than in the females at the differentiated stage (83 dph; *P* < 0.05). The *cldn11a* and *tnn* expressions were also significantly higher in the males than in the females at the differentiating stage (57 dph;* P* < 0.05). The *cldn11a* expression was significantly higher in the males than in the females at the undifferentiated stage (41 dph;* P* < 0.05).

### Sex steroid synthesis-related gene expression patterns

**Figure 3:**
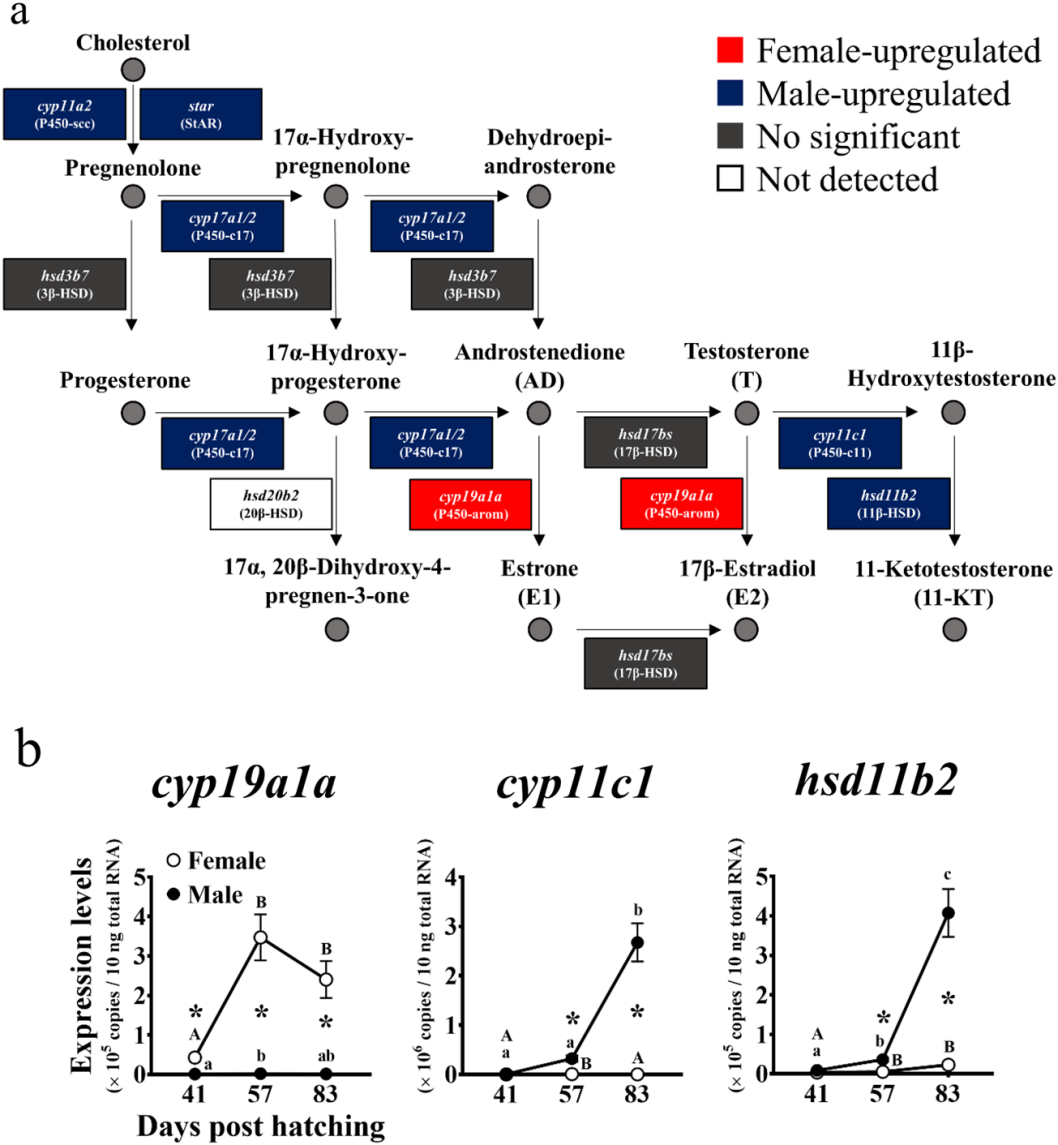
(**a**) Expression patterns of sex steroid biosynthesis-related genes in gonads of Pacific bluefin tuna at the sex differentiated stage. The diagram shows gonadal sex steroid biosynthesis pathways in fish^26,27^. Upregulated genes in females and males are indicated by red and blue boxes, respectively (*P* < 0.05). Genes not significantly different between sexes are indicated by gray boxes (*P* > 0.05). The gene with no detectable expression is indicated by a white box. Expression patterns of genes encoding 17β-hydroxysteroid dehydrogenases (17β-HSDs) are shown in Supplementary Fig. S1. (**b**) Temporal expression patterns of genes encoding key enzymes in estrogen and androgen biosynthesis during gonadal sex differentiation in Pacific bluefin tuna. Total RNA extracted from gonads in each sex at morphologically sex-undifferentiated (41 days post-hatching (dph)), differentiating (57 dph), and differentiated (83 dph) stages^6^ subjected to quantitative real-time reverse transcription PCR. Open and closed circles indicate genotypic females and males, respectively. Bars indicate mean ± standard error of the mean (SEM) (*n* = 3 fish). Different letters indicate significant differences (*P* < 0.05, one-way ANOVA followed by Tukey’s multiple comparisons test). Asterisks indicate significant differences between sexes at each age (*P* < 0.05, Welch’s *t*-test). *Cyp19a1a* expression data were previously reported^1^.

A comparative transcriptome analysis disclosed sexual dimorphism in sex steroid synthesis-related gene expression (Fig. 3a). *Star* (steroidogenic acute regulatory protein, StAR; contig No. g8869), *cyp11a2* (cytochrome P450 cholesterol side-chain cleavage enzyme, P450-scc; contig No. g1984), *cyp17a1* (cytochrome P450 17α-hydroxylase/C17-C20 lyase, P450-c17; contig No. g4434), *cyp17a2* (P450-c17; contig No. g26576), *cyp11c1* (cytochrome P450 family 11 subfamily C polypeptide 1, P450-c11; contig No. g16537), and *hsd11b2* (11β-hydroxysteroid dehydrogenase, 11β-HSD; contig No. g28927) were significantly upregulated in the males (*P* < 0.05). *Aromatase* (contig No. g2700) was significantly upregulated in the females (*P* < 0.05). There were no significant differences between sexes in terms of their *hsd3b7* (3β-hydroxysteroid dehydrogenase, 3β-HSD; contig No. g17096) and *hsd17bs* (17β-hydroxysteroid dehydrogenase, 17β-HSDs; see Supplementary Fig. S1 online) expression levels (*P* > 0.05). *Hsd20b2* (20β-hydroxysteroid dehydrogenase, 20β-HSD; contig No. g14056) was not expressed in either sex. Furthermore, there were no significant differences in the expressions of estrogen receptor encoding genes, *esr1* (*estrogen receptor 1*; contig No. g5109), *esr2a* (*estrogen receptor 2a*; contig No. g8921), and *esr2b* (*estrogen receptor 2b*; contig No. g22496) between sexes (*P* > 0.05; see Supplementary Table S3 and Fig. S2 online).

The qRT-PCR identified the temporal expression of the genes encoding estrogen and androgen biosynthesis-related enzymes (*aromatase*, *cyp11c1*, and *hsd11b2*) during gonadal sex differentiation (Fig. 3b). *Aromatase* expression data have been previously reported^1^. In the females, the *aromatase* expression significantly increased from the morphologically sex-undifferentiated (41 days post-hatching (dph)) to the differentiating (57 dph) stages (*P* < 0.05) and remained high until the differentiated stage (83 dph). In the males, however, the *aromatase* expression remained low until the differentiated stage. The *Aromatase* expression was significantly higher in the females than in the males at all stages (41–83 dph; *P* < 0.05). In the males, the *cyp11c1* expression significantly increased from the differentiating (57 dph) to the differentiated (83 dph) stages (*P* < 0.05). In the females, however, it remained low until the differentiated stage. In the males, the *hsd11b2* expression significantly increased from the undifferentiated (41 dph) to the differentiating (57 dph) stages (*P* < 0.05) and increased further until the differentiated (83 dph) stage (*P* < 0.05). In the females, however, it remained low until the differentiated stage. The *cyp11c1* and *hsd11b2* expression levels were significantly higher in the males than in the females at the differentiating (57 dph) and differentiated (83 dph) stages (*P* < 0.05).

### Sex differentiation-related gene expression patterns

**Figure 4:**
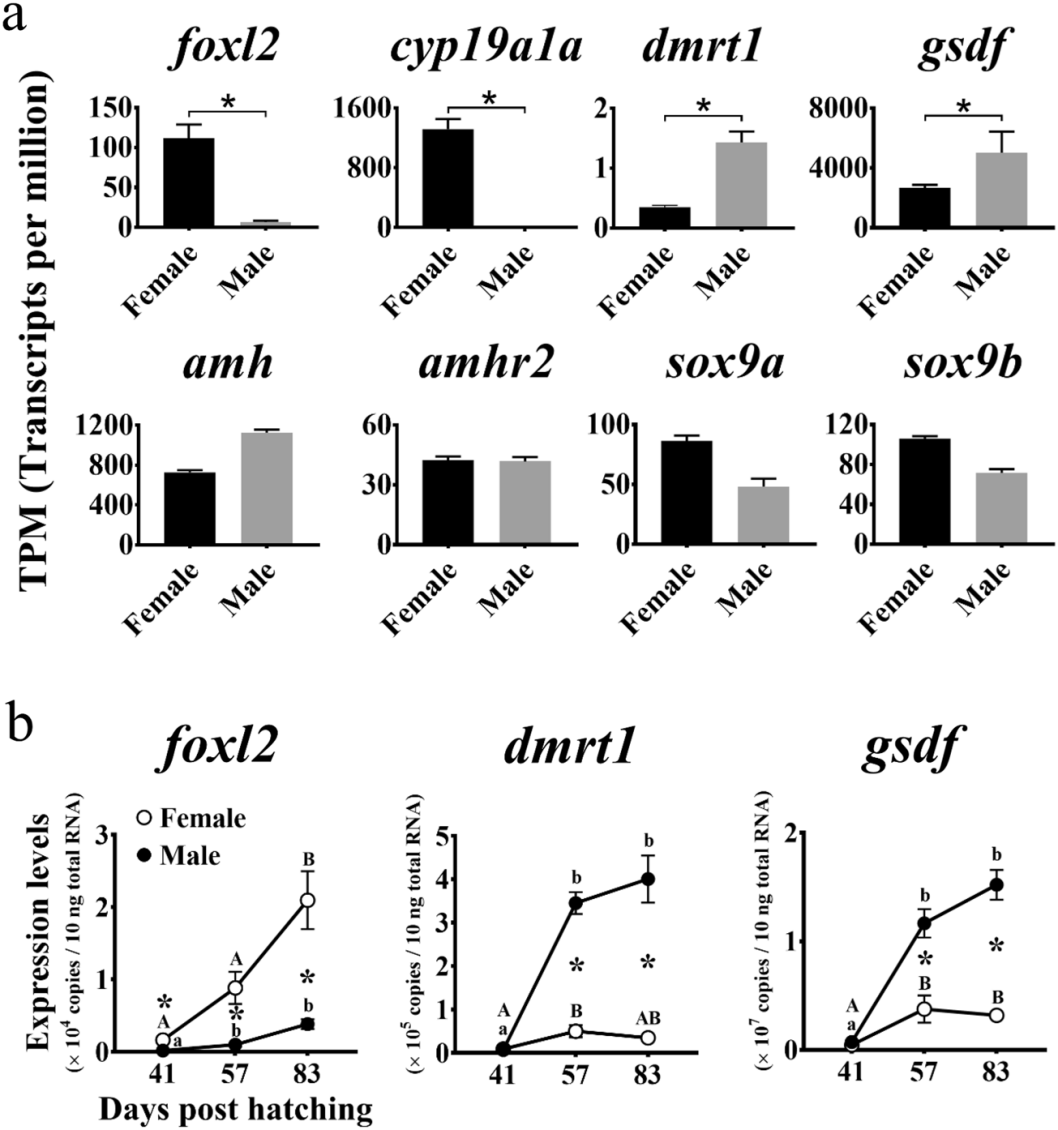
(**a**) Sex differentiation-related gene expression patterns in Pacific bluefin tuna gonads at the sex-differentiated stage. Expression levels correspond to transcripts per million (TPM). Statistical differences indicated by asterisks are false discovery rate-adjusted *P*-values (*P* < 0.05). (**b**) Temporal expression patterns of genes identified via comparative transcriptome analysis as candidate gonadal sex differentiation regulators in Pacific bluefin tuna. Total RNA extracted from the gonads of each sex at morphologically sex-undifferentiated (41 days post-hatching (dph)), differentiating (57 dph), and differentiated (83 dph) stages^6^ were subjected to quantitative real-time reverse transcription PCR. Open and closed circles indicate genotypic females and males, respectively. Bars indicate mean ± standard error of the mean (SEM) (*n* = 3 fish). Different letters indicate significant differences (*P* < 0.05, one-way ANOVA followed by Tukey’s multiple comparisons test). Asterisks indicate significant differences between sexes at each age (*P* < 0.05, Welch’s *t*-test).

A comparative transcriptome analysis revealed sexual dimorphism in sex differentiation-related gene expression (Fig. 4a). *Foxl2* (contig No. g17853) and *aromatase* were significantly upregulated in the females (*P* < 0.05), whereas *dmrt1* (contig No. g7920) and *gsdf* (contig No. g4772) were significantly upregulated in the males (*P* < 0.05). There were no significant differences between sexes in terms of their *amh* (contig No. g11182), *amhr2* (anti-Müllerian hormone receptor type 2; contig No. g370), *sox9a* (SRY-box transcription factor 9a; contig No. g15913), and *sox9b* (contig No. g14928) expression levels (*P* > 0.05).

The qRT-PCR identified the temporal expression of the sex differentiation-related genes with sexually dimorphic expression during gonadal sex differentiation (Fig. 4b). In the females, the *foxl2* expression significantly increased from the differentiating (57 dph) to the differentiated (83 dph) stages (*P* < 0.05). In the males, however, the *foxl2* expression remained low until the differentiated stage. The *Foxl2* expression was significantly higher in the females than in the males at all stages (41–83 dph; *P* < 0.05). In the males, the *dmrt1* and *gsdf* expression levels significantly increased from the undifferentiated (41 dph) to the differentiating (57 dph) stages (*P* < 0.05) and remained high until the differentiated stage (83 dph). In the females, however, the *dmrt1* and *gsdf* expression levels remained low until the differentiated stage. The *dmrt1* and *gsdf* expression levels were significantly higher in the males than in the females at the differentiating (57 dph) and the differentiated (83 dph) stages (*P* < 0.05).

### Sex steroid synthesis- and sex differentiation-related gene expression in sex-reversed gonads

**Figure 5:**
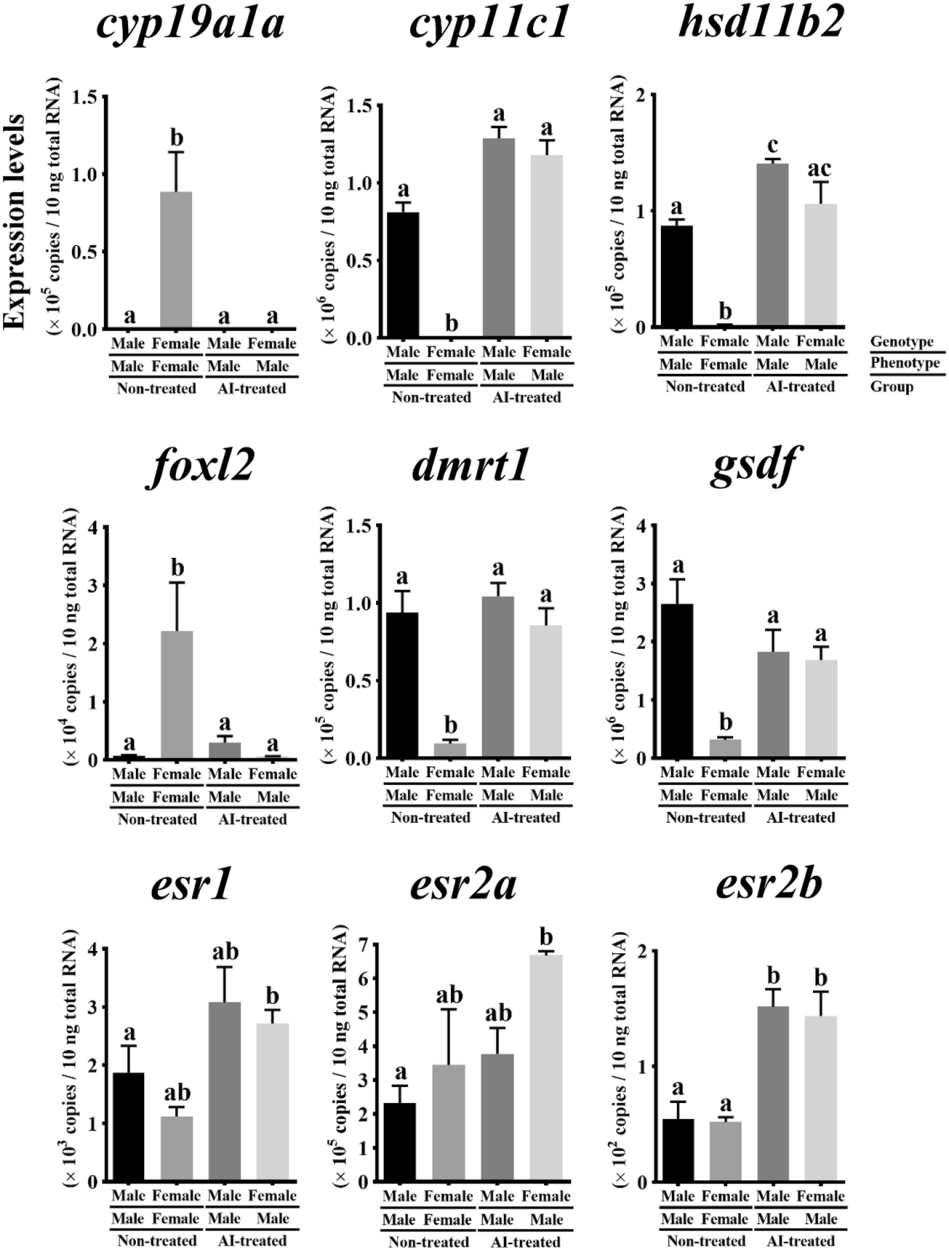
Expression patterns of sex steroid biosynthesis- and sex differentiation- related genes in sex-reversed (genotypic female/phenotypic male) Pacific bluefin tuna gonads. Genotypic female gonads were artificially masculinized via aromatase inhibitor administration^1^. Total RNA extracted from gonads at the morphologically sex-differentiated stage (70 days post-hatching) for each sex genotype in aromatase inhibitor-treated and untreated groups were subjected to quantitative real-time reverse transcription PCR. Bars indicate mean ± standard error of the mean (SEM) (*n* = 3 fish). Different letters indicate significant differences (*P* < 0.05; two-way ANOVA followed by Tukey’s multiple comparisons test).

Figure 5 shows the sex steroid synthesis- and sex differentiation-related gene expression levels in sex-reversed gonads (genotypic female/phenotypic male) artificially induced by the AI treatment^1^. The *aromatase* and *foxl2* expression levels were significantly higher in the genotypic/phenotypic females than the others (*P* < 0.05). The *cyp11c1*, *hsd11b2*, *dmrt1*, and *gsdf* expression levels in the genotypic female/phenotypic males were comparable to those in the genotypic/phenotypic males in both the AI-treated and untreated groups (*P* > 0.05). Nevertheless, the *cyp11c1*, *hsd11b2*, *dmrt1*, and *gsdf* expression levels were significantly lower in the genotypic/phenotypic females than the others (*P* < 0.05). No significant differences in the *esr1*, *esr2a*, and *esr2b* expressions were observed between the sexes in each treated group (*P* > 0.05). The expression levels of the estrogen receptors were higher in the AI-treated group than in the non-treated group. Furthermore, *esr2b* expression were significantly higher in the AI-treated group than those in the non-treated group (*P* < 0.05).

## Discussion

In this study, we initially updated our draft genome assembly for PBT. The completeness scores indicate that the current polished genome was slightly improved compared to the previous version^25^. Thereafter, we transcriptomically characterized gonadal sex differentiation in PBT using a reference gene dataset predicted from the polished draft genome. A comparative transcriptome analysis based on RNA-Seq identified 19,548 genes expressed in the PBT gonads at the sex-differentiated stage. Of these, 522 and 281 genes were upregulated in females and males, respectively. The qRT-PCR validated the expression profile and revealed the temporal expression patterns of the upregulated genes during gonadal sex differentiation in PBT. qRT-PCR validation targeted key factors in sex steroidogenesis and conservative factors and unreported factors in fish sex differentiation, which were enriched in multiple GO pathways. The expression profile of genes encoding key enzymes in estrogen and androgen biosynthesis, *aromatase* and *cyp11c1* were also validated through in situ hybridization (see Supplementary Fig. S3 online). Hence, this study provides numerous candidate genes potentially responsible for gonadal sex differentiation in PBT.

The enriched GO terms further characterized the upregulated genes for each sex. The genes upregulated in the females were highly enriched in cell/tissue development-related GO terms. These genes may be linked to ovarian cavity and lamellae formation because somatic cells proliferate in the female gonads to form them during ovarian differentiation^28,29^. We previously established the formation of these structures during ovarian differentiation in PBT^6^. By contrast, several upregulated genes in the males were enriched in structural development-related GO terms. These genes may be linked to the development of a structural framework, namely, the seminiferous epithelium, which is required for spermatogenesis in testes. The seminiferous epithelium is composed of Sertoli and germline cells^30,31^. The Sertoli cells create several junctions to provide developing germ cells with essential structural support^30,31^. Thus, our enrichment analysis disclosed numerous candidate genes responsible for developing ovarian and testicular structures in PBT.

Sex hormones play vital roles in gonadal sex differentiation in fish^10^. In this study, we found that the genes upregulated in the males were enriched in steroidogenesis-related GO terms. Furthermore, certain genes encoding key enzymes implicated in gonadal sex steroidogenesis, including *star*, *cyp11a2*, *cyp17a1*, *cyp17a2*, *cyp11c1*, and *hsd11b2*, were upregulated in the males. *Cyp11c1* and *hsd11b2* encode key enzymes in androgen biosynthesis, and qRT-PCR confirmed that they were upregulated in differentiating male gonads. We previously confirmed that the males have a higher plasma androgen (11-ketotestosterone) concentration than the females at the gonadal sex-differentiated stage^1^. Our results indicate that androgen biosynthesis is upregulated in the male gonads during testicular differentiation in PBT. Exogenous androgens, such as 17-α-methyltestosterone, induce masculinization in various species of fish with the female genotype^22^. However, the roles of endogenous androgens in testicular differentiation in fish are poorly understood. Androgen biosynthesis is absent in differentiating male Nile tilapia gonads^13,14^. Therefore, endogenous androgens may not be essential for testicular differentiation in this species. In contrast, androgen biosynthesis is upregulated in differentiating male rainbow trout gonads^15,16^. Nevertheless, it only occurs after early testicular differentiation^32^. Hence, testicular differentiation may be initiated in the absence of endogenous androgens in this species. In male PBT gonads, *cyp11c1* and *hsd11b2* were upregulated only after the onset of morphological sex differentiation. Li et al.^12^ suggested that endogenous androgens maintain testicular fate by suppressing ovarian differentiation in fish. In PBT, the upregulation of androgen biosynthesis may not directly induce testicular differentiation; rather, it might indirectly promote testicular differentiation by suppressing ovarian differentiation.

Unlike androgens, endogenous estrogens are essential for ovarian differentiation in fish^10–12^. We previously demonstrated *aromatase* upregulation and active estrogen biosynthesis in differentiating female PBT gonads^1^. We also showed that AI administration induced a sex reversal from genotypic female to phenotypic male^1^. The preceding results suggest that active aromatase-catalyzed estrogen biosynthesis is crucial for ovarian differentiation in PBT^1^, as reported in many fish species^10–12^. In the current study, our comparative transcriptome analysis revealed *foxl2* upregulation in the females. This gene is also critical in ovarian differentiation, which regulates the *aromatase* transcription either by directly binding its promoter or interacting with nuclear receptor subfamily 5 group A member 1 (Nr5a1)^10–12^. The qRT-PCR analysis disclosed that *foxl2* was upregulated mainly in differentiating female gonads. Notably, both *foxl2* and *aromatase* were more highly upregulated in the females than in the males at the morphologically sex-undifferentiated stage. Similar to prior reports on other fish species^11,12^, our results suggested that Foxl2 also plays a central role in ovarian differentiation in PBT by upregulating *aromatase*. Furthermore, downregulation of the genes controlling testicular differentiation was observed in *foxl2-*knockout genotypic female Nile tilapia^33^. Foxl2 may also contribute to ovarian differentiation in PBT by suppressing the genes regulating testicular differentiation. Future studies should be undertaken to confirm the role for Foxl2 in ovarian differentiation in tuna, particularly whether it transcriptionally regulates *aromatase* and the genes promoting testicular differentiation.

We isolated two genes from the *foxl2* family in PBT genome, *foxl2* (*foxl2a*) and *foxl3* (*foxl2b*; contig No. g15820). *Foxl3*, a paralog of *foxl2*, is essential for female germ cell fate decisions in medaka^34^ and Nile tilapia^35^. In this study, comparative transcriptome analysis detected no *foxl3* expression in both sex PBT gonads at the sex-differentiated stage. Furthermore, no differences in germ cell development-related gene expression, including *vasa* (contig No. g25316), *dead end* (contig No. g24023), *nanos1* (contig No. g6249 and g10448), *nanos2* (contig No. g7592 and 7600), *sycp3* (*synaptonemal complex protein 3*; contig No. g29093), and *dmc1* (*DNA meiotic recombinase 1*; contig No. g15865) between sexes were observed (see Supplementary Tables S1 and 3 online). Our previous analysis revealed that PBT germ cells develop in a sexually dimorphic manner after sex differentiation: germ cell proliferation and differentiation occur earlier in the ovaries than in the testes^6^. Gene expressions in germ cells inducing sexual dimorphic development are expected to occur after gonadal sex differentiation in PBT. Notably, two contigs were predicted as genes encoding *nanos1* and *nanos2* from the updated PBT genome. In contrast, no contig was predicted as *nanos3*. Further studies are required to understand the existence of nanos family genes in the PBT genome.

Our comparative transcriptome analysis and qRT-PCR revealed equal expression levels of estrogen receptor encoding genes, *esr1*, *esr2a*, and *esr2b*, between sexes in the sex-differentiated stage PBT gonads, similar to the previous observations in several fish species, such as rainbow trout^36^ and Nile tilapia^14^. The *esr2a* expression level was relatively high compared with those of *esr1* and *esr2b*, suggesting that Esr2a may primarily mediate estrogens in female PBT gonads during ovarian differentiation, although its role in male gonads during sex differentiation remains unclear.

20β-HSD plays a pivotal role in final oocyte maturation by producing MIH, i.e., 17α, 20β-dihydroxy-4-pregnen-3-one^26,27^. In our comparative transcriptome analysis, no *hsd20b2* expression was detected in either sex. This is a reasonable result because no oocytes were observed in the sex-differentiated stage PBT ovaries subjected to RNA-seq.

The transcription factor Dmrt1 is implicated in vertebrate testicular differentiation^2^. Furthermore, Gsdf promotes testicular differentiation in fish^10^. Our comparative transcriptome analysis identified *dmrt1* and *gsdf* upregulation in the male PBT. Our qRT-PCR analysis revealed *dmrt1* and *gsdf* upregulation in both differentiating male and masculinized female (genotypic female/phenotypic male) gonads. The foregoing results and our understanding of the important roles of Dmrt1 and Gsdf in fish testicular differentiation^10^ suggest that Dmrt1 and Gsdf regulate this developmental process in PBT.

AMH signaling comprises Amh and its receptor Amhr2, and it has a vital function in testicular differentiation in several fish species^10^. Furthermore, several studies have suggested that Sox9s (two types of *sox9* genes are found in fish, namely, *sox9a* and *sox9b* (or *sox9a2*), because of teleost-specific genome duplication^37^, whereas mammals possess only a single copy of *sox9*) may participate in gonadal sex differentiation in fish^32,38^. Nevertheless, our comparative transcriptome analysis showed that *amh*, *amhr2*, *sox9a*, and *sox9b* were expressed at the same levels in both sexes. AMH signaling and Sox9s are generally regarded as gonadal development factor and sex differentiation regulators in fish^10^. In PBT, these factors may be involved in ovarian and testicular development rather than gonadal sex differentiation.

In fish gonads, sexually dimorphic expression of the genes regulating sex differentiation generally occurs before morphological sex differentiation^10^. Our previous^1^ and current studies confirmed *aromatase* and *foxl2* upregulation in female PBT gonads at the morphologically sex-undifferentiated stage (41 dph). By that time, gonadal sex differentiation had already been initiated at the gene expression level. In contrast, the expression levels of *dmrt1* and *gsdf* did not differ between the sexes at this stage. We speculate that Foxl2 and aromatase triggering ovarian differentiation, whereas Dmrt1 and Gsdf are not required for triggering testicular differentiation. However, they are vital for promoting testicular differentiation. Incidentally, our qRT-PCR analysis identified upregulation of *cldn11a* in male PBT gonads at the morphologically sex-undifferentiated stage (41 dph). In mice, *claudin 11* expresses in Sertoli cells and is an obligatory protein for forming tight junctions in testis^39^. *Cldn11a* may be an upstream factor that initially induces testicular differentiation in fish. To the best of our knowledge, this is the first report on the relation of *cldn11a* to the early gonadal sex differentiation in fish. This gene will be studied in detail in the future.

We recently identified a male-specific homolog of *sulfotransferase family 1, cytosolic sulfotransferase 6* (*sult1st6y*; contig No. g29404), as a candidate sex-determining gene in PBT^25^. Sulfotransferases deactivate endogenous estrogens by sulfating them^40^. Our comparative transcriptome analysis revealed *sult1st6y* upregulation in the males (see Supplementary Table S2 online). Furthermore, we previously demonstrated that inhibiting estrogen biosynthesis via AI treatment induces testicular differentiation in female gonads^1^. We speculated that Sult1st6y would trigger testicular differentiation through estrogen deactivation in male gonads at the onset of gonadal sex differentiation^25^, and estrogen deficiency induces upregulation of the genes promoting testicular differentiation, particularly *dmrt1* and *gsdf*. We are currently investigating *sult1st6y* expression patterns and the mechanism by which Sult1st6y deactivates estrogens.

In conclusion, we have developed a global gene expression profile for gonadal sex differentiation in PBT using NGS technology. Based on our findings, we propose that ovarian differentiation is mainly induced by aromatase and Foxl2, whereas Dmrt1 and Gsdf play central roles in testicular differentiation in PBT. We previously demonstrated the morphological characteristics of gonadal sex differentiation and the vital role of aromatase-mediated estrogen synthesis in this developmental process in PBT^1,6^. Overall, the discoveries made herein lay theoretical and empirical foundations for understanding the sex differentiation mechanisms in PBT and other tuna species.

## Methods

### Ethics statement

All experiments were conducted in accordance with the Guidelines for the Care and Use of Live Fish of the Fisheries Technology Institute (FTI) of the Japan Fisheries Research and Education Agency (FRA) and the ARRIVE guidelines. All experiments were approved by the Institutional Animal Care and Use Committee of FTI.

### Draft genome improvement and protein coding sequence prediction

The draft genome sequence for male PBT consisted of 948 scaffolds and was previously published in 2021^25^. This sequence was polished with PolishCLR^41^ (https://github.com/isugifNF/polishCLR) using the Pacific Biosciences (PacBio) and Illumina data from the previous version. Genome completeness was evaluated and compared against that of the previous version using BUSCO^42^ v. 5.3.0 (https://gitlab.com/ezlab/busco/-/releases#5.3.0) and the Actinopterygii ortholog set. The protein-coding sequences were predicted with reference to those from the PBT genome published in 2013^23^, the genome of Southern bluefin tuna (*Thunnus maccoyii*) in the GenBank^43^ (https://www.ncbi.nlm.nih.gov/genbank/), and five model fish genomes in the Ensembl database^44^ (release 104) (https://www.ensembl.org/index.html?redirect=n0) including zebrafish, stickleback (*Gasterosteus aculeatus*), medaka, tiger puffer (*Takifugu rubripes*), and tetraodon (*Tetraodon nigroviridis*). The foregoing protein sequences were mapped to the current PBT genome with Exonerate^45^ v. 2.4.0 (https://anaconda.org/bioconda/exonerate). All prediction results were merged excluding the overlapping loci with lower alignment scores. The predicted protein sequences were compared with those of two model fishes, zebrafish and medaka (Ensembl database, Release 104), using the BLASTP program (E-value < 1.0E^−4^).

### Sample collection and RNA extraction and sequencing

PBT gonads at the sex-differentiated stage (Fig. 1a) were collected at 100 dph from fish reared from fertilized eggs. The oogonia and type-A spermatogonia were solitarily distributed in the differentiated ovary and testis. The total length and body weight of all PBTs sampled were 25.6 ± 11.5 cm and 258.4 ± 94.5 g (*n* = 10 fish, mean ± standard error of the mean (SEM)), respectively. Dissected gonads were stored in RNAlater (Ambion, Austin, TX, USA) at − 30 °C until RNA extraction. The sex phenotypes and genotypes of each fish were determined via histological observation and PCR-based genotypic sex identification, respectively^6,25^. All fish gonads differentiated into ovaries or testes according to their sex genotypes.

Total RNA was extracted from the gonads, and RNA extracts were treated with DNase using the RNeasy Mini kit (Qiagen, Hilden, Germany) according to the manufacturer’s instructions. The concentration and quality of purified total RNA were determined using NanoDrop One (Thermo Fisher Scientific, Waltham, MA) and the Agilent 2200 TapeStation System with RNA screen tape (Agilent Technologies, Santa Clara, CA). High-quality RNA extracts with RNA integrity number (RIN) > 7 were selected from three fish per sex (Table 1). The cDNA libraries were constructed using 1 µg total RNA and Illumina Stranded mRNA Prep (Illumina, San Diego, CA) according to the manufacturer’s instructions. The libraries were sequenced into 75-bp paired end reads using NextSeq 500 systems (Illumina). The raw data were demultiplexed, trimmed of adapters, and converted into the FASTQ format using bcl2fastq v. 2.20 (Illumina).

### Comparative transcriptome analysis

RNA-Seq data analysis was performed using the CLC Genomics Workbench v. 12.0.3 (QIAGEN GmbH, Hilden, Germany). Raw reads were trimmed with default parameters (quality limit = 0.05 and ambiguous limit = 2), and the trimmed reads were mapped onto the reference sequences with default parameters (mismatch cost = 2, insertion cost = 3, deletion cost = 3, length fraction = 0.8, and similarity fraction = 0.8). The read count files containing the values of the reads mapped against the reference sequences were then loaded into the integrated Differential Expression and Pathway analysis (iDEP) web platform^46^ (http://bioinformatics.sdstate.edu/idep/). The data were then normalized using DEseq2^47^ (https://bioconductor.org/packages/release/bioc/html/DESeq2.html). The DESeq2 program in iDEP was also used to identify differentially expressed genes between sexes. The false discovery rate and minimum fold change cutoff/threshold values were set to 0.05 and 2, respectively. The differentially expressed genes were then subjected to enrichment analysis in the GO biological process database using the Ensembl zebrafish gene IDs (Release 104) in iDEP.

### qRT-PCR

Previously sampled PBT gonads^1,6^ were stored in RNAlater (Ambion) at − 30 °C until RNA extraction. Total RNA extraction and qRT-PCR were performed as previously described^1^. The qRT-PCR reaction volumes contained 0.4 μM of each forward and reverse gene-specific primers, except for *anxa1b*, which contained 0.1 μM of each forward and reverse primer. The gene-specific primers and PCR amplification conditions are shown in Supplementary Table S6. The primers were designed using CLC Main Workbench v. 8.0.1 (QIAGEN GmbH). The quantification standard was a plasmid containing the partial cDNA sequence of a target gene. Standard seven-point sets ranged from 1 × 10^8^ to 1 × 10^2^ copies and were prepared using tenfold serial dilutions. Technical duplicates were performed for all experimental samples and standards. The intra-assay coefficient of variation (CV) was determined using repeated standard sample measurements (*n* = 8) (see Supplementary Table S6 online). The expression levels are presented as means ± SEM.

### Statistical analysis

For the comparative transcriptome analysis, statistical analyses were conducted in iDEP. For the temporal gene expression comparison using qRT-PCR, the statistical analyses included Welch’s *t*-test (between sexes at each sampling period) and one-way analysis of variance (ANOVA), followed by Tukey’s multiple comparisons test (for the expression trends in each sex). Statistical analyses of the gene expression levels in the sex-reversed gonads included two-way ANOVA followed by Tukey’s multiple comparisons test. Statistical analyses of the qRT-PCR data were performed in GraphPad Prism v. 7.0 (GraphPad Software, San Diego, CA, USA). Statistically significant differences were determined at* P* < 0.05.

### Supplementary Information

Supplementary Information 1.

Supplementary Figures.

Supplementary Tables.

## Data Availability

The data that support the findings of this study are available from the corresponding author upon reasonable request. The polished genome sequences were deposited in the DNA Data Bank of Japan (DDBJ) under Accession Numbers BOUD02000001–BOUD02000580. The RNA sequencing results were deposited in the DDBJ Sequence Read Archive (DRA) under Accession Numbers DRR458350–DRR458355.
