# Supplementary material for: Transcriptome characterization of gonadal sex differentiation in Pacific bluefin tuna, Thunnus orientalis (Temminck et Schlegel)
Source: Sci Rep. 2023 Aug 24;13:13867. doi: 10.1038/s41598-023-40914-y (PMC10449831; doi:10.1038/s41598-023-40914-y)
Supplement: Supplementary file 2 — Supplementary Figures. [file 41598_2023_40914_MOESM2_ESM.docx]

**Supplementary Figure S1.** Expression pattern of genes encoding 17β-hydroxysteroid dehydrogenases (17β-HSDs) in sex-differentiated stage Pacific bluefin tuna gonads. Expression levels correspond to transcripts per million (TPM). There are no significant differences in the 17β-HSDs-encoding gene expression levels between sexes (*P* > 0.05).

**Supplementary Figure S2.** Expression pattern of genes encoding estrogen receptors in sex-differentiated stage Pacific bluefin tuna gonads. Expression levels correspond to transcripts per million (TPM). There are no significant differences in the estrogen receptor-encoding gene expression levels between sexes (*P* > 0.05).


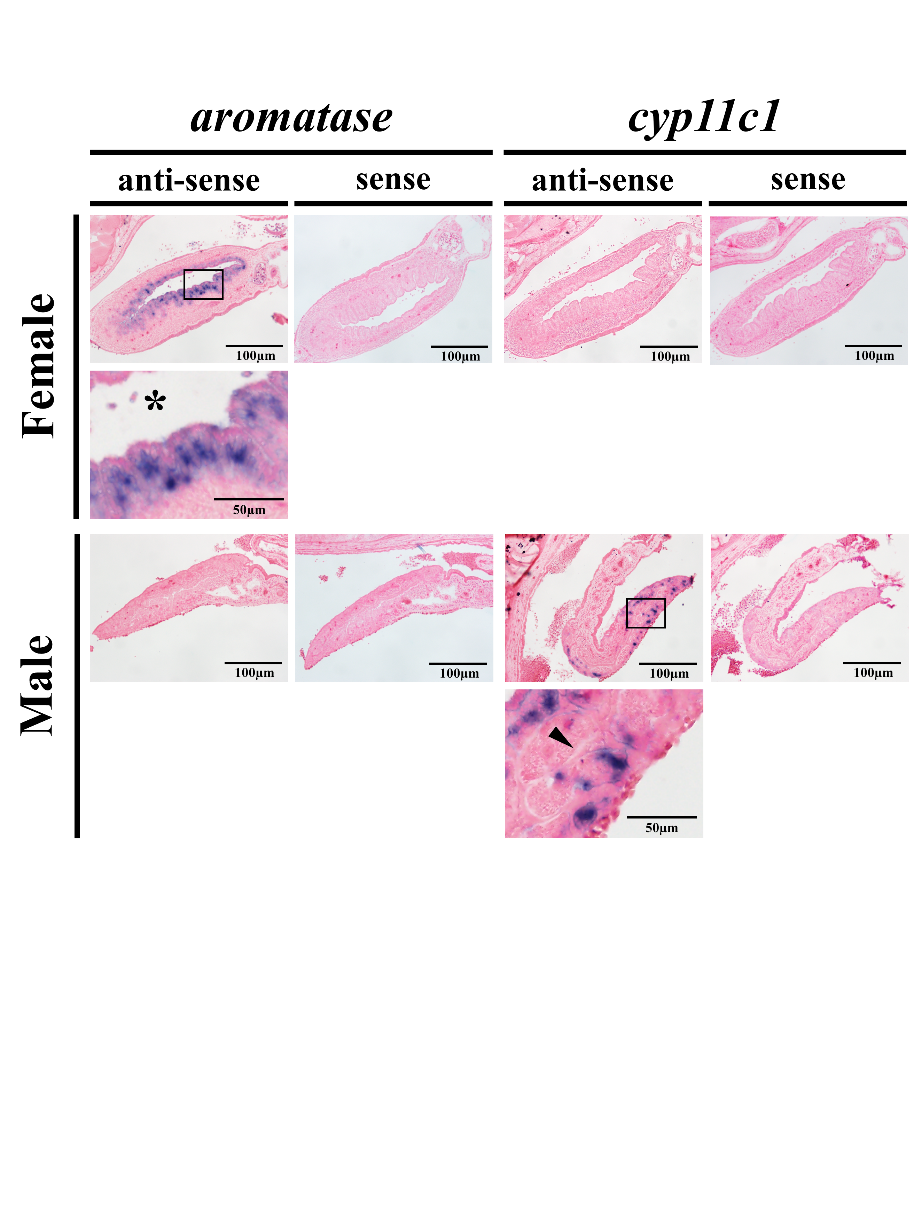


**Supplementary Figure S3.** *In situ* hybridization analysis of genes encoding key enzymes implicated in estrogen and androgen biosynthesis, *aromatase* (aromatase) and *cyp11c1* (P450-c11) in the sex-differentiated stage (83 days post-hatching) Pacific bluefin tuna gonad. Sequential sections hybridized with digoxigenin (DIG)-labeled antisense and sense probes. RNA probes were transcribed *in vitro* using DIG-labeled uridine triphosphate (Roche, Mannheim, Germany) and SP6 or T7 RNA polymerase (Roche). Gene expressions were detected via a chromogenic reaction using Nitro-Blue Tetrazolium chloride (NBT)/ 5-Bromo-4- Chloro-3’-Indolylphosphatase p-Toluidine salt (BCIP) (Roche) as alkaline phosphatase substrate. After color development, sections were counterstained using Nuclear Fast Red (Vector Laboratories, CA, USA). The asterisk and arrowhead indicate the ovarian cavity and the efferent duct, respectively.
